# ARPC1A correlates with poor prognosis in prostate cancer and is up-regulated by glutamine metabolism to promote tumor cell migration, invasion and cytoskeletal changes

**DOI:** 10.1186/s13578-023-00985-w

**Published:** 2023-02-22

**Authors:** Ye-Hui Chen, Hang Chen, Ting-Ting Lin, Jun-Ming Zhu, Jia-Yin Chen, Ru-Nan Dong, Shao-Hao Chen, Fei Lin, Zhi-Bin Ke, Jin-Bei Huang, Yong Wei, Qing-Shui Zheng, Xue-Yi Xue, Ning Xu

**Affiliations:** 1 Department of Urology, The First Affiliated Hospital, Urology Research Institute, Fujian Medical University, 20 Chazhong Road, Fuzhou, 350005 China; 2 Fujian Key Laboratory of Precision Medicine for Cancer, The First Affiliated Hospital, Fujian Medical University, Fuzhou, 350005 China

**Keywords:** ARPC1A, Prostate cancer, Cytoskeleton formation, Glutamine metabolism

## Abstract

**Objective:**

This study aimed to identify potential biomarkers for prostate cancer (PCa) progression and metastasis, and to discern their biological functions.

**Methods:**

Bioinformatics methods were used to screen for hub genes. The expression level of key hub genes in PCa was determined and their prognostic significance was examined. A series of functional assays were performed to investigate the function of the highest-ranking hub gene.

**Results:**

Actin related protein 2/3 complex subunit 1A (ARPC1A) was identified as the hub gene. ARPC1A was highly expressed in PCa tissues and cell lines, and was an independent prognostic factor for predicting biochemical recurrence after radical prostatectomy and overall survival of PCa patients. Knockdown of ARPC1A inhibited PCa cell migration, invasion and cytoskeleton formation, but had no impact on cell proliferation and cell cycle progression. In vivo, ARPC1A overexpression promoted lung metastasis of PCa, but had no efffect on tumor growth. Additionally, glutamine metabolism was identified as an upstream regulator of ARPC1A, and promoted migration, invasion and cytoskeletal changes of PCa cell through ARPC1A.

**Conclusion:**

These findings suggested that ARPC1A, which correlates with poor prognosis in PCa, functions downstream of glutamine metabolism to regulate cytoskeletal changes, cellular migration and cellular invasion in this disease.

**Supplementary Information:**

The online version contains supplementary material available at 10.1186/s13578-023-00985-w.

## Background

Prostate cancer (PCa) is the fifth principal cause of death and also the second most frequent cancer in males worldwide [1, 2]. PCa is a heterogeneous disease; the majority of men experience an indolent form of the disease, while others experience a highly aggressive form that will ultimately metastasize [3]. The 5-year survival rate for men diagnosed with indolent PCa is more than 90%, while for patients with metastatic disease this is less than 30% [4, 5]. Although a growing number of functional studies have led to a deeper understanding of the pathogenesis of PCa, reliable biomarkers for disease risk and prognosis are still lacking [6]. Therefore, the identification of biomarkers that can differentiate between PCa patients that will develop indolent and aggressive forms of this cancer is urgently needed to improve the accuracy of disease diagnosis and prognosis, and therefore the effective management of patients with this disease.

A cancer biomarker is defined as a substance or a process that is indicative of the presence of cancer in the body. Bioinformatics analysis provides an appealing tool for identifying potential biomarkers for diseases. Such analysis allows researchers to delve into integrated data sets of numerous clinical samples from different independent studies, providing a data infrastructure both for discovering potential biomarkers and for improving our understanding about various cancers [7, 8]. Weighted gene co-expression network analysis (WGCNA) is an algorithm that construct free-scale gene co-expression networks to explore the relationships between gene sets and clinical features. It has been successfully used to screen biomarkers and therapeutic targets for PCa in recent studies [1, 9–11].

The aim of this study was to identify potential biomarkers related to the progression and metastasis of PCa and to examine their biological functions. Our bioinformatics analysis identified actin related protein 2/3 complex subunit 1A (ARPC1A) as a key hub gene of interest associated with PCa. We have confirmed ARPC1A overexpression in PCa tissues and also its role in cellular processes associated with tumor invasion and spread. Importantly, we have shown that ARPC1A has prognostic value for patients with PCa.

## Materials and methods

### Patients and clinical specimens

Twenty pairs of cancerous and matched adjacent noncancerous tissue specimens were obtained from patients diagnosed with PCa between October 2019 and March 2020 in First Affiliated Hospital of Fujian Medical University. Samples used to construct the tissue microarray (TMA) were collected from 301 patients with detailed clinical information who had undergone radical prostatectomy (RP) between January 2011 and December 2018 in our hospital. The clinicopathological characteristics of these patients are shown in Table 1. All patients were followed up until March 2020, and the follow-up period ranged from 1.2 to 9 years after surgery. This study was approved by the Ethics Committee of our hospital. All patients signed informed consent forms before sample collection.

**Table 1:** Clinicopathological characteristics of 301 patients with PCa from TMA cohort

Clinicopathological characteristic	Value
Age, years	
Mean ± SD	63.54 ± 7.63
Range	42–85
BMI, kg/m^2^	
Mean ± SD	23.21 ± 3.26
Range	16.23–31.12
T Stage, n (%)	
T2	140 (46.5)
T3	128 (42.5)
T4	33 (11.0)
N Stage, n (%)	
N0	160 (53.2)
N1	141 (46.8)
M Stage, n (%)	
M0	273 (90.7)
M1	28 (9.3)
Gleason grade group, n (%)	
≤ 6	24 (8.0)
3 + 4	40 (13.3)
4 + 3	82 (27.2)
8	83 (27.6)
≥ 9	72 (23.9)
Diagnostic PSA,ng/ml, n (%)	
< 10	18 (6.0)
10–20	204 (67.8)
> 20	79 (26.2)
Positive surgical margin, n (%)	
Yes	142 (47.2)
No	159 (52.8)
Adjuvant therapy, n (%)	
Yes	161 (53.5)
No	140 (46.5)
ARPC1A expression, n (%)	
(±)	93 (30.9)
( +)	114 (37.9)
(+ +)	56 (18.6)
(+ + +)	38 (12.6)

*P < 0.05

### Construction of the co-expression network and analysis of the relationship between gene modules and disease

**Fig. 1:**
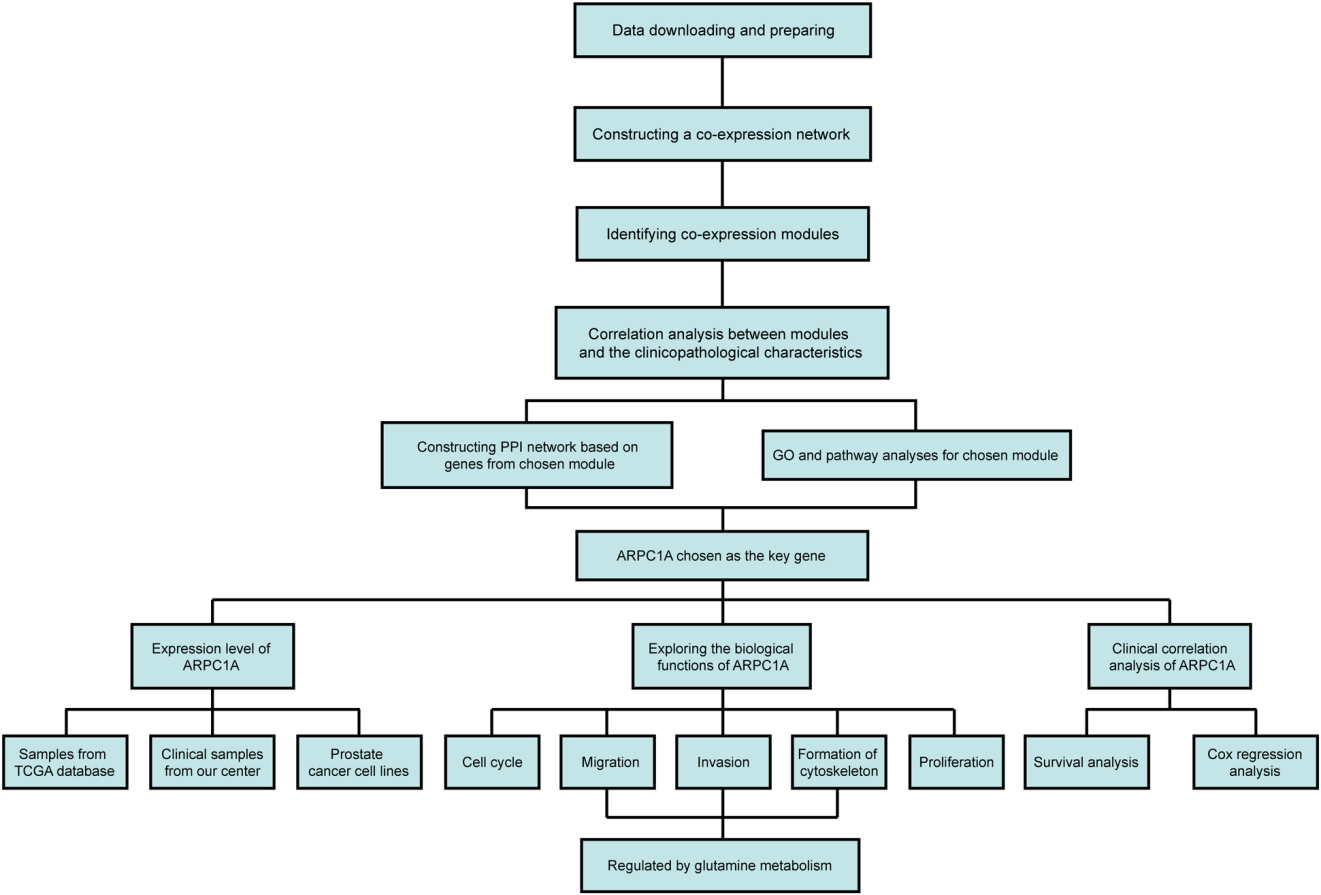
Flowchart detailing the study design

The workflow of our study is shown in Fig. 1. We downloaded mRNA expression profiles of PCa patients from the cancer genome atlas (TCGA) database (https://portal.gdc.cancer.gov/). After data cleaning and quality checking, 434 cases of PCa were finally included in this study [12]. The clinicopathological characteristics of these cases are summarized in Table 2. WGCNA was conducted to explore the relationship between gene modules and disease progression. Gene significance (GS) was defined as the log10 transformation of the *P*-value (GS = lg*P*) in the linear regression between gene expression and clinical features. Module significance (MS) was defined as the average GS for all genes in a module. The module with the highest absolute MS among all the selected modules for a given clinical feature was considered to be the one related to that feature. For each gene module, the expression patterns of all genes were summarized into a single characteristic expression value, the module eigengene (ME), which was used as the major component in the principal component analysis. We calculated the correlations between MEs and clinical features to identify the most relevant gene module for each given clinical feature. The module that demonstrated the strongest correlation with clinical features of interest was selected for further analysis.

**Table 2:** Clinicopathological characteristics of 434 patients with PCa from TCGA database

		ARPC1A expression		
Variables	N	Low	High	*P* value
Total, n (%)	434	185 (42.6)	249 (57.4)	
Age, n (%)				0.336
< 60 years	178 (41.0)	71 (38.4)	107 (43.0)	
≥ 60 years	256 (59.0)	114 (61.6)	142 (57.0)	
T Stage, n (%)				< 0.001*
T2	157 (36.2)	104 (56.2)	53 (21.3)	
T3	256 (59.0)	76 (41.1)	180 (72.3)	
T4	21 (4.8)	5 (2.7)	16 (6.4)	
N Stage, n (%)				0.007*
N0	291 (67.1)	137 (74.1)	154 (61.8)	
N1	143 (32.9)	48 (25.9)	95 (38.2)	
M Stage, n (%)				0.763
M0	404 (93.1)	173 (93.5)	231 (92.8)	
M1	30 (6.9)	12 (6.5)	18 (7.2)	
Gleason Score, n (%)				0.094
≤ 6	25 (5.8)	14 (7.6)	11 (4.4)	
7	91 (21.0)	45 (24.3)	46 (18.5)	
≥ 8	301 (73.2)	126 (68.1)	192 (77.1)	
Race, n (%)				0.346
White	359 (82.7)	159 (86.0)	200 (80.4)	
Yellow	26 (6.0)	11 (5.9)	15 (6.0)	
Black	7 (1.6)	2 (1.1)	5 (2.0)	
Unknown	42 (9.7)	13 (7.0)	29 (11.6)	
Adjuvant therapy, n (%)				0.283
Yes	155 (35.7)	62 (33.5)	93 (37.3)	
No	103 (23.7)	40 (21.6)	63 (25.4)	
Unknown	176 (40.6)	83 (44.9)	93 (37.3)	

*P < 0.05

### Functional enrichment analysis

WebGestalt (http://www.webgestalt.org/) and Metascape (http://metascape.org/) are online databases providing a comprehensive set of functional annotation tools for researchers to better understand biological meaning behind large lists of genes [13]. We uploaded genes in the chosen module to perform gene ontology (GO) analysis and pathway enrichment analysis. A *P*-value < 0.05 was considered statistically significant. Gene set enrichment analysis (GSEA) software was used to explore biological functions related to the candidate gene. The cut-off criteria for GSEA were a nominal *P*-value < 0.05 and a false discovery rate (FDR) < 0.25.

### Protein–protein interaction (PPI) network construction and hub gene selection

Search Tool for the Retrieval of Interacting Genes (STRING) is a biological database for constructing protein–protein interaction (PPI) networks, providing a system-wide view of interactions between each member [14]. Genes of selected modules were mapped to STRING to explore their relationships with each other, and a combined score > 0.4 was set as the cut-off criterion, as described previously [15]. Subsequently, a PPI network was constructed using Cytoscape software. The Centiscape plug-in was used to search for the most important nodes in a network by calculating centrality parameters for each node [15]. The genes with highest degree of connectivity were selected as the hub genes.

### Cell lines and cell culture

Four human PCa cell lines, PC-3, DU-145, 22Rv1, and LNCaP, were purchased from the American Type Culture Collection (ATCC, Manassas, VA, USA). The BPH-1 human prostate epithelial cell line was purchased from the Procell Life Technology Company (Wuhan, China). All cell lines were authenticated by short tandem repeat genotype analysis. Cells were cultured in RPMI-1640 medium (Gibco, Grand Island, NY, USA) with 10% bovine serum at 37 °C in a humidified atmosphere containing 5% CO_2_.

### Short interfering RNA (siRNA) and ARPC1A knockdown

Three siRNAs were designed for ARPC1A knockdown. The sequences were as follows: 5′-ACGGACACAUCACAGGUAUUGTT-3′, 5′-GCGAUUUCAUUCCAUUCUUGATT-3′ and 5′-GCCUAUGUCUGGAGUCAGAAATT-3′. Cells were seeded in 6-well plates (Corning Costar, Corning, USA) at a density of 3 × 10^5^ cells per well. When the cells had reached 70% confluency, they were transfected with siRNA using Lipofectamine 3000 reagent (Life Technologies, Carlsbad, CA, USA) according to the manufacturer’s protocol. After verifying the efficiency of knockdown, two efficient siRNAs were selected for subsequent experiments.

### Establishment of stable cell lines overexpressing ARPC1A

Lentivirus for ARPC1A overexpression and control lentivirus were purchased from GeneChem Corporation (Shanghai, China). Transfection was performed according to the manufacturer’s instructions. Virally-transduced cells were cultured for at least one week in the presence of puromycin (2 μg/ml; Gibco) to select stable clones. At the indicated time points, the cells were harvested for subsequent experiments.

### Quantitative real-time polymerase chain reaction (qRT-PCR)

Total RNA was extracted from PCa cell lines and tissue specimens using TRIzol reagent (Invitrogen, Carlsbad, CA, USA) according to the manufacturer’s instructions. Complementary DNA was synthesized using the PrimeScript RT reagent kit (Takara, Osaka, Japan). qRT-PCR was performed using the SYBR Green assay (Takara) and an ABI 7500 Real-Time PCR System (Applied Biosystems, Waltham, USA). Primer sequences used were as follows: ARPC1A, forward primer: 5′-ATTGCCCTCAGTCCCAATAATCA-3′, reverse primer: 5′-CAAGTGACAATGCGGTCGC-3′; Glyceraldehyde-3-phosphate dehydrogenase (GAPDH), forward primer: 5′-TGACTTCAACAGCGACACCCA-3′, reverse primer: 5′-CACCCTGTTGCTGTAGCCAAA-3′.

### Western blotting (WB)

Total protein extracts from tissues and cells were prepared using RIPA lysis buffer (Beyotime, Haimen, China) supplemented with PMSF (Beyotime). Sample protein concertation was measured using the bicinchoninic acid kit (Beyotime). Proteins were separated on 8%–12% polyacrylamide gels by electrophoresis and subsequently transferred onto polyvinylidene difluoride membranes (Merck Millipore, Darmstadt, Germany). The membranes were blocked with 5% nonfat milk for 1 h at room temperature and then incubated with primary anti-ARPC1A (1:1000; Abcam, Cambridge, UK) or anti-GAPDH (1:2000; Abcam) antibodies overnight at 4 °C. After washing with Tris-buffered saline/0.1% Tween 20 (TBST), the membranes were incubated with horseradish peroxidase-conjugated goat anti‐rabbit IgG antibody (1:5000; MDL Biotechnology, Beijing, China) for 2 h at room temperature and immunoreactive bands then detected using an enhanced chemiluminescence reagent.

### Immunohistochemistry

Immunohistochemistry was performed on the TMA according to the manufacturer’s recommended protocols. Briefly, the TMA slides were deparaffinized and rehydrated, and then endogenous tissue peroxides were quenched by incubation with 0.3% H_2_O_2_ for 30 min. For antigen retrieval, the slides were boiled in sodium citrate buffer (10 mmol/L, pH 6.0) in a pressure cooker for 7 min. Subsequently, nonspecific binding was blocked with 5% normal goat serum, and then the slides were incubated with primary anti-ARPC1A antibody (1:200, Abcam) overnight at 4 °C, and then with anti-rabbit secondary antibody (Zhongshan Biotech, Zhongshan, China). Immunoreactivity was visualized using diaminobenzidine as the chromogen substrate. The slides were counterstained with hematoxylin, then dehydrated and mounted with glass coverslips according to a standard laboratory protocol. A previously described semi-quantitative analysis method based on staining intensity and the proportion of positive cells was used to derive the total ARPC1A immunostaining score[16]. Staining intensity (I) was recorded as 0, absent; 1, weak; 2, moderate; 3, strong, whereas the proportion (P) of positive cells was recorded as 0, < 5%; 1, 5% to 25%; 2, 26% to 50%; 3, 51% to 75%; and 4, > 75%. A score for each histological grade (H-score) was determined as the summary of intensity and proportion H-score = I × P according to a previous study. The final ARPC1A expression score was calculated using the value of the percent positivity score multiplied by the staining intensity score as “-” (score, 0–1), “ + ” (score, 2–3), “ +  + ” (score, 4–5), and “ +  +  + ” (score ≥ 6) (Fig. 4A).

### Cell proliferation, migration and invasion assays

Cell proliferation was evaluated using a CCK-8 assay Kit (Dojindo, Kumamoto, Japan) according to the manufacturer’s protocol. Briefly, cells were seeded in 96-well plates (Corning Costar) at a density of 3 × 10^3^ cells per well and cultured for 24, 48, 72, 96 or 120 h. CCK-8 reagent (10 μl) was added to each well and the cells then incubated for 2 h before the absorbance at 450 nm was measured using a SpectraMax M5 microplate reader (Molecular Devices, Sunnyvale, CA, USA).

The migration and invasion of cells were evaluated using a Transwell assay (Corning Costar). Briefly, 3 × 10^4^ cells resuspended in 2000 μl of serum-free medium were added to the upper chamber of a Transwell system with an 8 μm pore membrane. The chamber was uncoated (for the migration assay) or coated with Matrigel (BD Biocoat, Bedford, MA, USA; for the invasion assay). The lower chamber contained 300 μl medium containing 10% fetal bovine serum. Cells were allowed to migrate or invade for 24 h, and then the cells that had not penetrated the membrane were removed with a cotton swab. The cells on the lower surface of the membrane were subsequently fixed and stained, and cells then counted in five randomly selected fields under a light microscope.

Additionally, wound healing assays were conducted to evaluate cell migration. Cells were cultured for 24 h to achieve confluent monolayer cultures. A pipette tip was then used to manually scratch the cell monolayers to generate a uniform wound. We photographed the wounds at 0 h, 16 h, 24 h, and 48 h, then drew two parallel lines at the edges of the cells and measured the distance between them. Migration rate was used to assessed the the migration of cells quantitatively.

### Flow cytometry analysis

Cells were seeded in 6-well plates and cultured for 24 h. Next, cells were harvested and fixed with 70% ethanol at 4 °C overnight. The fixed cells were then washed, stained with propidium iodide and filtered through a 70-micron cell strainer immediately prior to analysis by flow cytometry, which was carried out on a FACScan flow cytometer (ACEA Biosciences, San Diego, CA, USA).

### Immunofluorescence analysis

Immunofluorescence analysis was performed according to the procedures described in a previous study [17, 18]. Cells were fixed in 4% paraformaldehyde, permeabilized in 0.25% Triton X-100, stained with Fluorescein isothiocyanate (FITC)‐conjugated phalloidin (1:100; MDL Biotechnology) to label the filamentous actin cytoskeleton, and then counterstained with 4,6‐diamidino‐2‐phenylindole to label cell nuclei (Invitrogen). The cells were then imaged using an epifluorescence microscope to observe the morphology of pseudopodia.

### In vivo experiments

A total of 26 male BALB/c nude mice (8 weeks, 20–22 g) were used in present study. To establish subcutaneous xenograft tumor model, 2 × 10^6^ PC-3 cells were injected on sides of the flank of mice (n = 8 per group). 28 days after injection, the mice were sacrificed the size and the weight of tumors were measured. To establish pulmonary metastasis model, 2 × 10^6^ PC-3 cells were inoculated via caudal vein (n = 10 per group). After 28 days, the fluorescent signal of pulmonary metastases was detected and analyzed using an IVIS Lumina II In Vivo Imaging System (Perkin Elmer) with Live Imaging Acquisition and Analysis software. Then the lungs of mice were removed and HE staining was performed to check the number of metastatic nodules. All the protocols of animal experiments have been approved by the Animal Care Committee of Fujian Medical University.

### Statistical analysists

Statistical analyses were conducted using SPSS version 22.0 software (SPSS, Chicago, IL, USA) and GraphPad 5.0 software (GraphPad Software, San Diego, CA, USA). Univariate and multivariate analyses were conducted using the Cox proportional hazard regression model. Survival curves were plotted using the Kaplan–Meier method and log-rank test. We used the “Surv_cutpoint” function of “Survival” R package (version 3.2.13, by Terry M Therneau, performed on R version 3.5.1) to get the best cutpoint of ARPC1A expression for the TMA cohort, which can maximize the difference of survival curves between subgroups. Comparison between two groups was performed using a Student *t* test. Comparison between paired samples was performed using a paired *t* test. *P* < 0.05 was considered statistically significant.

## Results

### Identification of a key module and gene associated with PCa progression

We extracted gene expression data and associated clinical information of PCa cases from the TGCA database. After a pre-processing clean-up and quality control stage, these data were subjected to WGCNA to explore associations between gene clusters and clinical traits associated with PCa. Based on the scale free topology, we selected a soft-thresholding power of β = 16 (scale free R^2^ = 0.93; Additional file 1: Fig. S1A, B). Additional file 1: Fig. S1C, D demonstrate the positive result of the rationality test. A sample dendrogram was then constructed based on the similarity among samples at the gene expression level; the dendrogram along with the associated clinical characteristics of these samples is shown in Additional file 1: Fig. S1E. Six gene modules were identified (Additional file 1: Fig. S1F). Among all modules, the turquoise module demonstrated both the highest MS for T stage (Additional file 1: Fig. S1G), as well as the strongest correlation between ME and T stage (Additional file 1: Fig. S1K). Additionally, the turquoise module demonstrated both the highest MS for N stage (Additional file 1: Fig. S1I), and also the strongest correlation between ME and N stage (Additional file 1: Fig. S1K). The turquoise module, which included 137 genes, was therefore identified as the module most relevant to tumor progression and lymph node metastasis in PCa (Additional file 1: Fig. S1H, J).

**Fig. 2:**
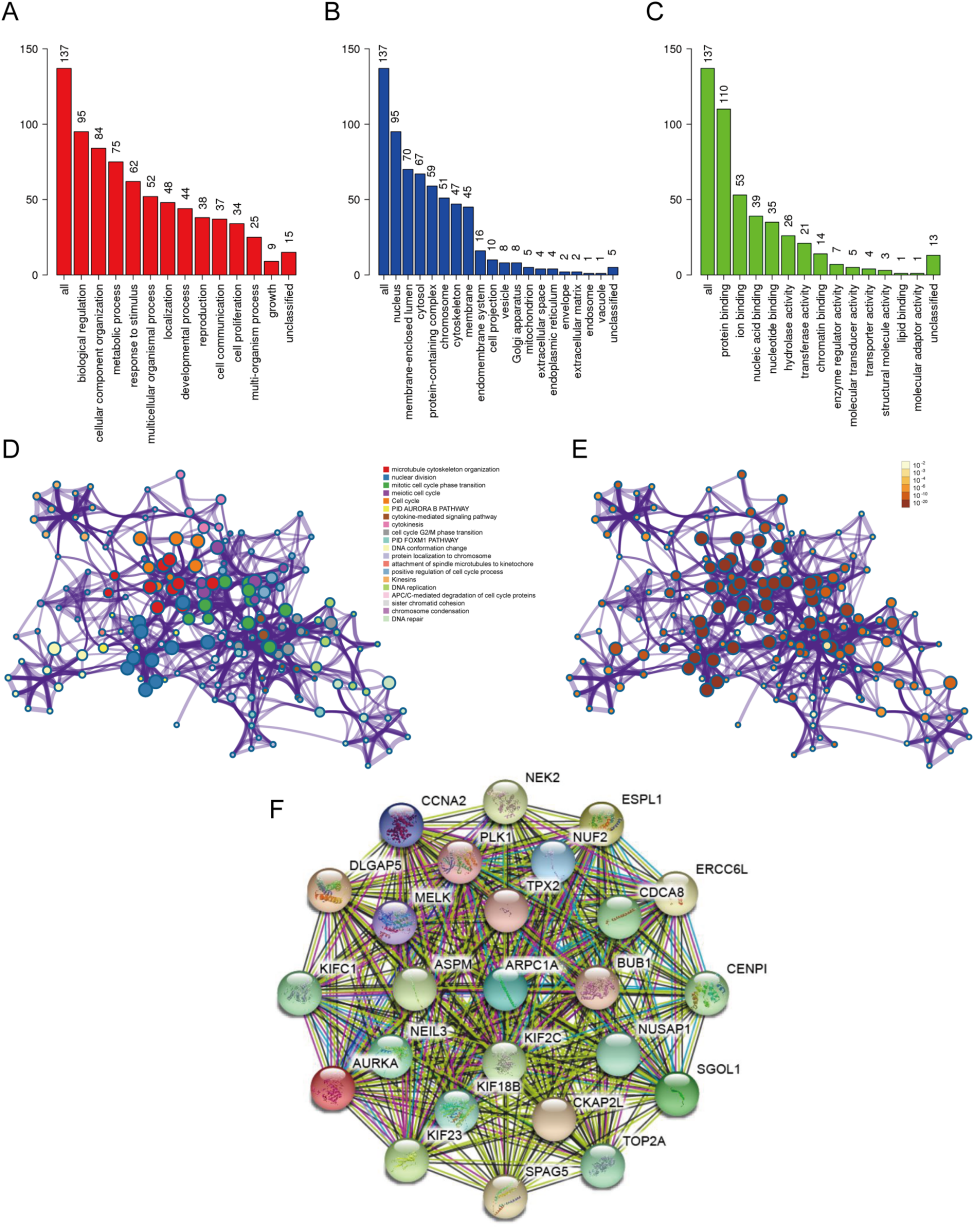
Functional enrichment analysis and construction of the PPI network. **A**–**C** GO analysis of **A** ‘biological process’, **B** ‘cellular component’, and **C** ‘molecular function’ for genes in the turquoise module. **D** Functional enrichment analysis for genes in turquoise module. **E** The *P*-value for each gene in the network. **F** PPI network constructed using the STRING database

Next, we performed functional enrichment analysis to look for the biological processes and pathways relevant to the turquoise module. GO analysis was conducted using the online WebGestalt tool. GO analysis of ‘biological process’ revealed that the genes in the turquoise module were mainly associate with the terms ‘biological regulation’, ‘cellular component organization’, ‘metabolic process’ and ‘response to stimulus’ (Fig. 2A). GO analysis of ‘cellular component’ showed that these genes were mainly enriched in the terms ‘nucleus’, ‘membrane-enclosed lumen’, ‘cytosol’ and ‘protein-containing complex’ (Fig. 2B). And GO analysis of ‘molecular function’ revealed that these genes were enriched in the terms ‘protein binding’, ‘ion binding’, ‘nucleic acid binding’ and ‘nucleotide binding’ (Fig. 2C).

Metascape was used to further investigate relevant biological processes and pathways. The results showed that the biological processes and pathways were mainly related to microtubule cytoskeleton organization, nuclear division, mitotic cell cycle phase transition, meiotic cell cycle, and cell cycle (Fig. 2D, E). A PPI network was then constructed using the STRING online database using a minimum required interaction score > 0.4 (Fig. 2F). Our analysis identified ARPC1A as the hub gene with the highest degree of connectivity and this gene was therefore selected for further investigation.

### ARPC1A is highly expressed in PCa tissues and cell lines, and correlates with poor patient prognosis

**Fig. 3:**
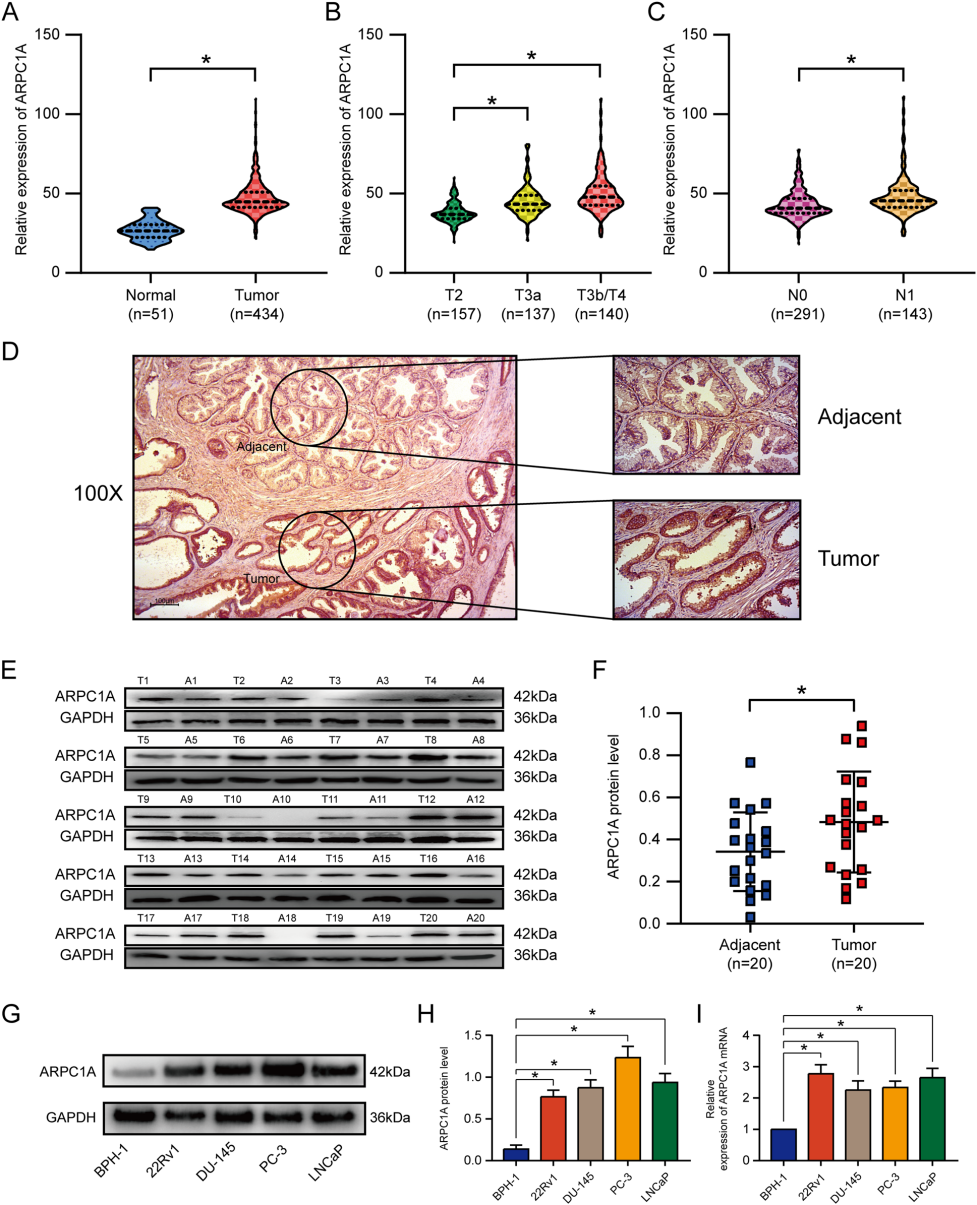
ARPC1A is highly expressed in PCa tissues and cell lines. **A**–**C** ARPC1A mRNA expression levels in PCa samples and normal prostate samples from the TCGA database. **D** Immunochemistry analysis of ARPC1A protein expression in PCa tissue and adjacent prostate tissue. **E**, **F** ARPC1A protein expression levels in 20 paired PCa tissues and adjacent prostate tissues from our center. **G**–**I** ARPC1A mRNA and protein expression in several PCa cell lines and a prostate epithelial cell line. *P < 0.05

Next, we sought to determine whether ARPC1A expression differed between PCa and normal prostate tissues. We examined data from the TCGA database that contained expression data for 434 PCa tissues and 51 normal prostate tissues. We found that ARPC1A expression was significantly higher in tumors than that in normal tissues (Fig. 3A), and was significantly associated with tumor progression and lymph node metastasis (Fig. 3B, C). In agreement with these findings, an analysis of 20 pairs of samples from our center revealed a similar result for ARPC1A protein levels (Fig. 3D–F). Next, we examined ARPC1A expression in human PCa cell lines and found it to be much higher in cancer lines (22Rv1, DU-145, PC-3 and LNCaP), when compared with BPH-1 normal human prostate epithelial cells (Fig. 3G–I).

**Fig. 4:**
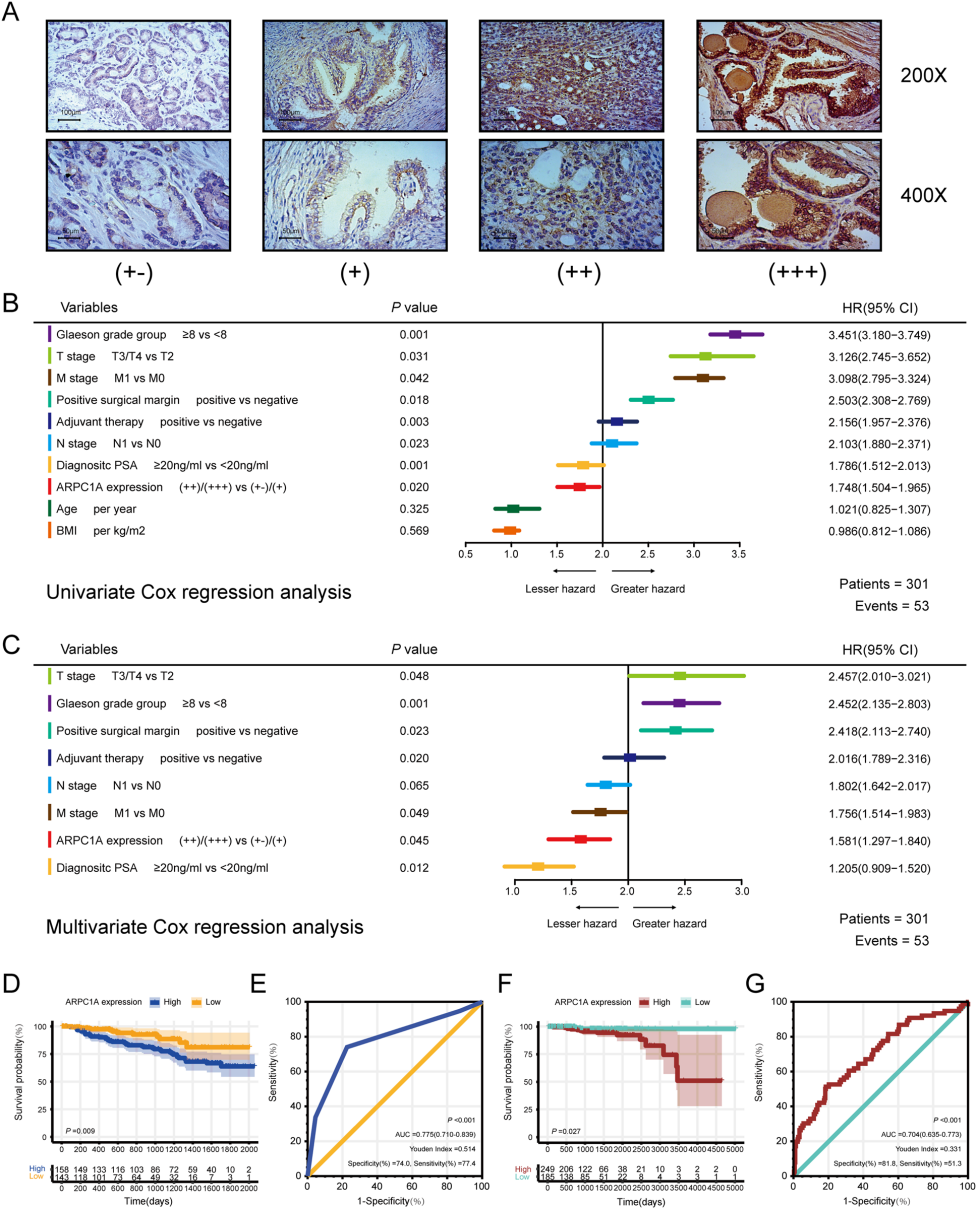
ARPC1A is associated with the poor prognosis of PCa patients. **A** Differences in ARPC1A protein expression among PCa tissue specimens. **B** Univariate Cox regression analysis for BCR after RP for patients with PCa. **C** Multivariate Cox regression analysis for BCR after RP for patients with PCa. **D** The cumulative incidence of BCR after RP in PCa patients with different ARPC1A expression levels from the TMA cohort. **E** ROC curve of ARPC1A expression levels demonstrates the potential of ARPC1A as a biomarker for predicting BCR after RP. **F** The cumulative incidence of death in PCa patients with different ARPC1A expression levels from the TCGA cohort. **G** ROC curve of ARPC1A expression levels demonstrates the potential of ARPC1A as a biomarker for predicting OS

We next investigated the relationship between ARPC1A expression and prognosis in PCa based on a TMA cohort (containing 301 cases) and the TCGA cohort (containing 434 cases). This analysis revealed that increased ARPC1A expression was associated with American Joint Committee on Cancer T stage and N stage in the TCGA cohort (Table 2). Both univariate and multivariate Cox regression analysis demonstrated that ARPC1A expression was an independent prognostic factor for biochemical recurrence (BCR) after RP for patients with PCa (Fig. 4B, C). Moreover, survival analysis, whose endpoint was BCR after RP, demonstrated that the BCR rate differed significantly between patients with high and low ARPC1A expression, based on the TMA cohort (Fig. 4D). Receiver operating characteristic (ROC) curve analysis was performed to evaluate the ability of ARPC1A to predict BCR for PCa patients. This analysis demonstrated that ARPC1A had considerable predictive value for BCR, with an area under the curve (AUC) of 0.775, a specificity of 74.0% and a sensitivity of 77.4% (Fig. 4E). Similarly, ARPC1A expression had significant predictive value in relation to overall survival (OS), based on patient samples from the TCGA cohort (Fig. 4F). For OS, the AUC value was 0.704, with a specificity of 81.8% and a sensitivity of 51.3% (Fig. 4G).

### ARPC1A promotes PCa migration, invasion and cytoskeleton formation, but not cell proliferation and cell cycle progression

**Fig. 5:**
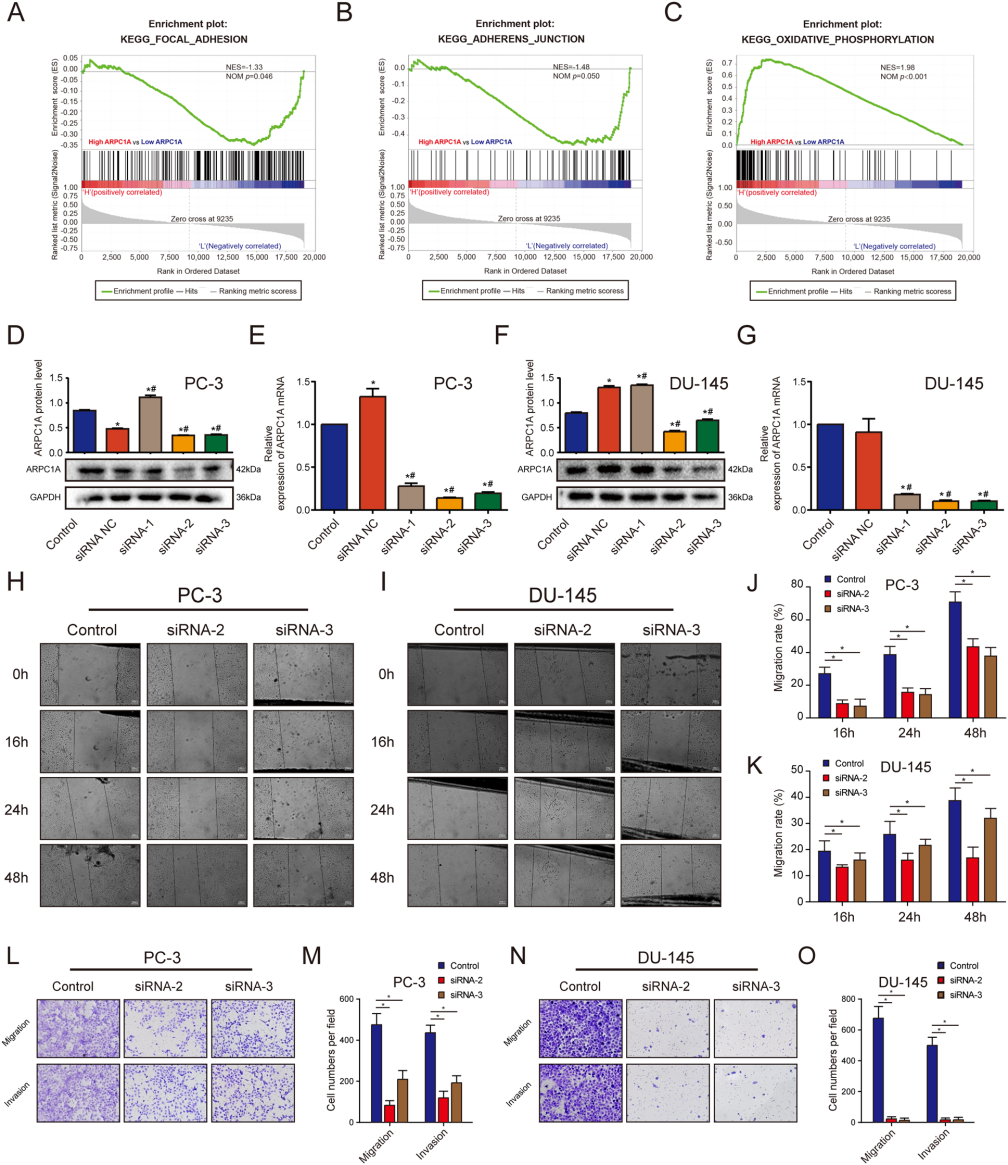
Knockdown of ARPC1A inhibits PCa cell migration and invasion in vitro. **A**–**C** The GSEA gene sets that have the strongest association with high ARPC1A expression in PCa cells. **D**–**G** WB and qRT-PCR analysis was performed on PC-3 and DU-145 cells to validate the silencing efficiency of siRNAs. *: *P* < 0.05, compared with control group; #: *P* < 0.05, compared with the siRNA NC group. (H–K) Differences in migration between control and ARPC1A siRNA-transfected PCa cells, as determined using a wound healing assay. *: *P* < 0.05. **L**–**O** Differences in migration and invasion between control and ARPC1A siRNA-transfected PCa cells as determined using Transwell assays. *: *P* < 0.05

**Fig. 6:**
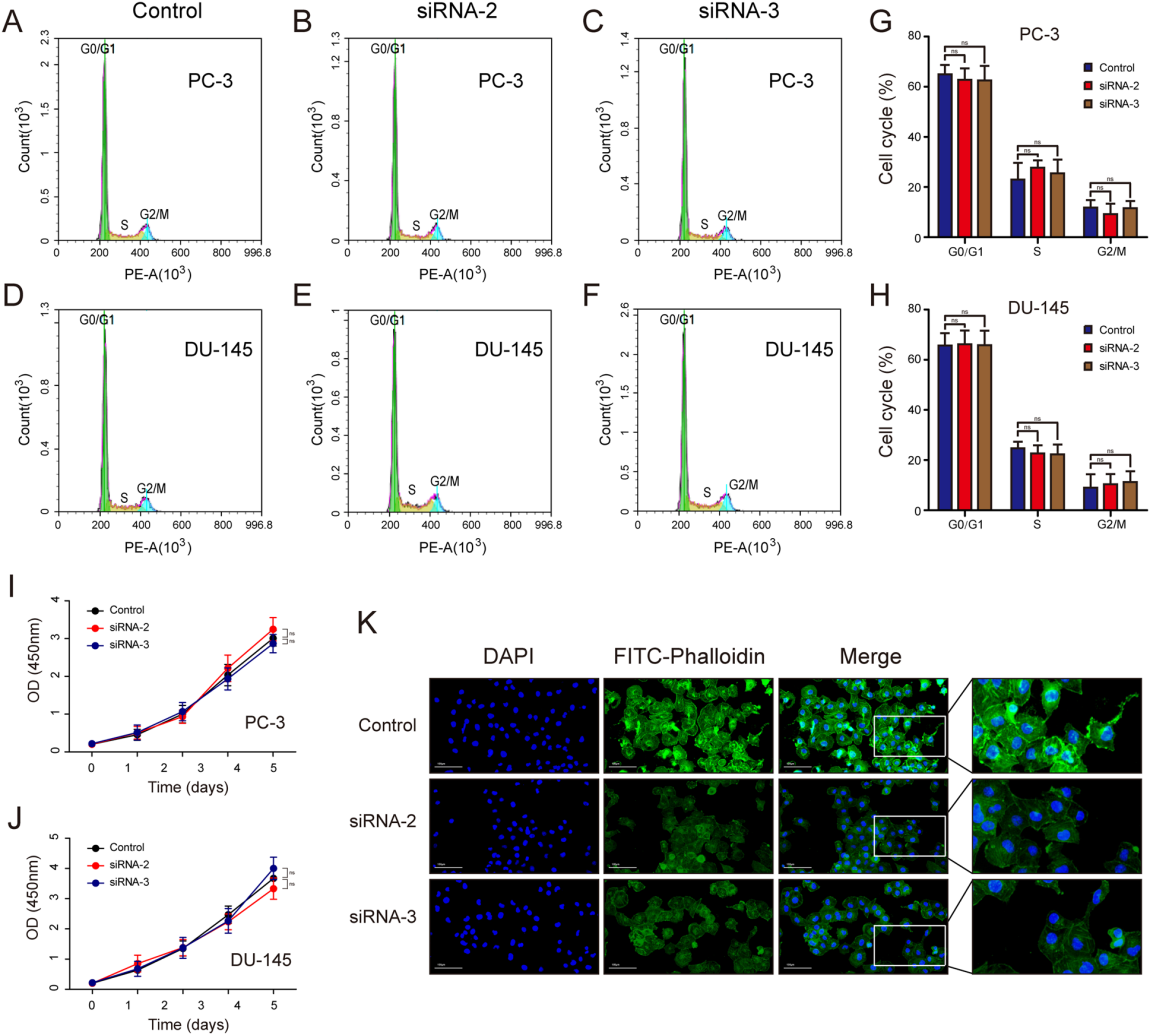
Knockdown of ARPC1A inhibits cytoskeleton formation, but has no impact on cell cycle progression and cell proliferation. **A**–**H** Differences in cell cycle stage between control and ARPC1A siRNA-transfected PCa cells, as determined by flow cytometry analysis. *: *P* < 0.05; ns: no statistical significance. **I**–**J** Differences in cell proliferation between control and ARPC1A siRNA-transfected PCa cells, as determined using a CCK-8 assay. *: *P* < 0.05; ns: no statistical significance. **K** Immunofluorescence analysis of the actin cytoskeleton and cellular morphology in control and ARPC1A siRNA-transfected PC-3 cells. *DAPI* 4’,6-diamidino-2-phenylindole, *FITC* fluorescein isothiocyanate

**Fig. 7:**
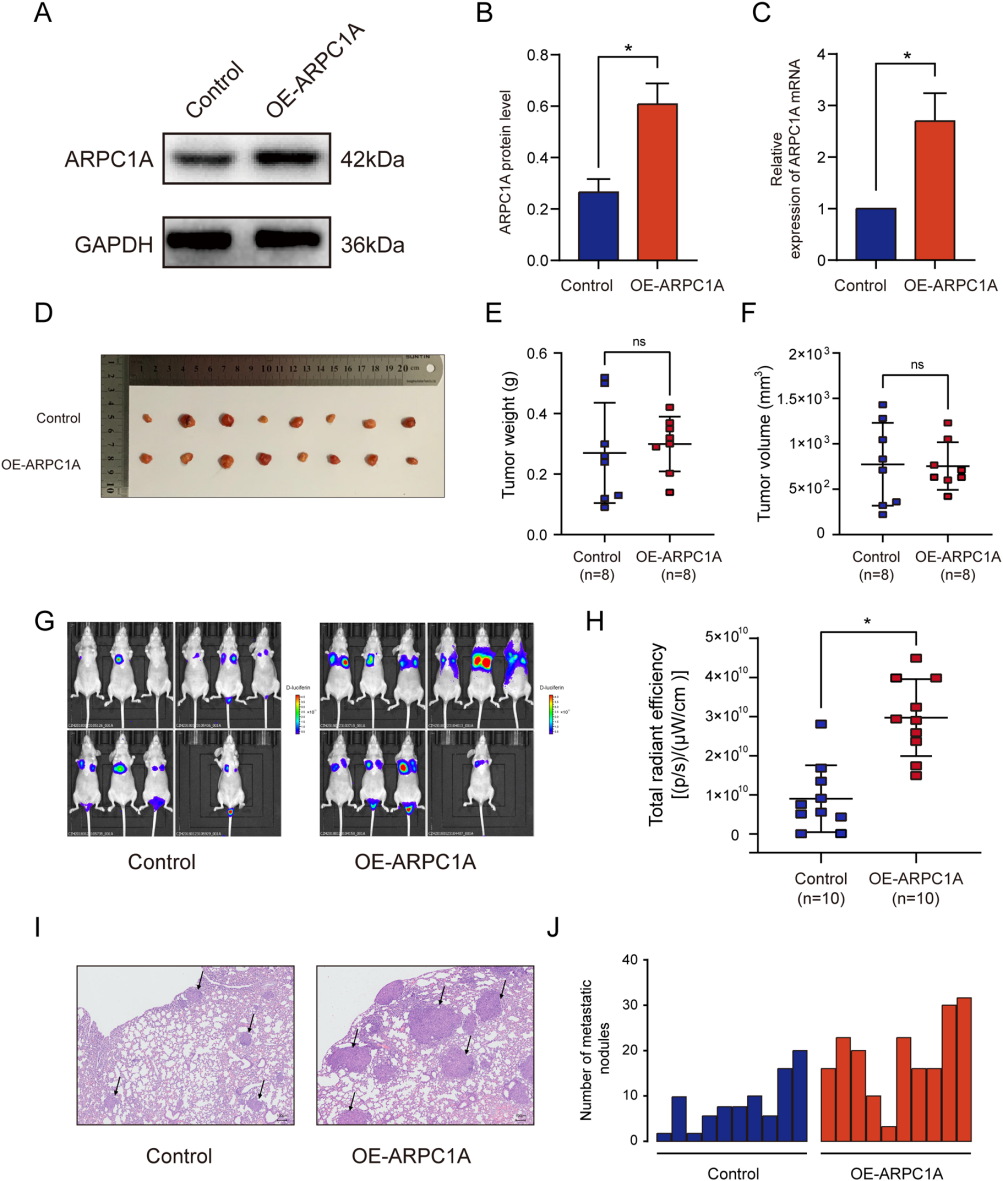
Overexpression of ARPC1A promotes lung metastasis of PCa, but has no effect on tumor growth. **A**–**C** WB and qRT-PCR were performed to verify ARPC1A overexpression in the ARPC1A stable PC-3 cell line. *: *P* < 0.05. **D**–**F** Subcutaneous xenograft tumor experiment was performed to evaluate the effect of ARPC1A on tumor growth, **E** the size and **F** the weight of xenograft tumors was measured. ns: no statistical significance. **G**–**J** Lung metastasis model was established to evaluate the effect of ARPC1A on tumor metastasis, **H** the fluorescent signal of lung metastases was detected and **I** the number of metastatic nodules was counted; **J** Representative HE staining of lung tissues. Arrowheads indicate the metastatic nodules. *: *P* < 0.05

To evaluate the biological role of ARPC1A in PCa, we performed gain- and loss-of-function studies in cultured PCa cells. Firstly, GSEA was performed to predict the biological processes most closely associated with ARPC1A expression. The analysis revealed that the terms ‘focal adhesion’, ‘adherens junction’ and ‘oxidative phosphorylation’ were the biological categories most related to ARPC1A (Fig. 5A–C). Of the three siRNAs evaluated in this study to suppress ARPC1A expression, siRNA-2 and siRNA-3 exhibited a high silencing efficiency, so were consequently used for the subsequent experiments (Fig. 5D–G). The impact of ARPC1A knockdown on the migration of PCa cells was assessed using wound healing and transwell migration assays. These assays demonstrated that ARPC1A silencing inhibited the migration of both PC-3 and DU-145 cells (Fig. 5H–O). A Matrigel-based transwell invasion assay was also performed to evaluate the effects of ARPC1A suppression on PCa cell invasion. The number of PCa cells that had passed through to the underside of the transwell membrane was significantly lower in ARPC1A siRNA group, when compared with the control group (Fig. 5L–O). However, ARPC1A silencing had no significant effect on cell cycle progression, in either PC-3 or DU-145 cells (Fig. 6A–H). A CCK-8 assay was used to investigate the impact of ARPC1A suppression on the proliferation of PCa cells, and revealed no significant difference between the cells of the control and ARPC1A siRNA groups (Fig. 6I, J). Phalloidin binds F-actin, which is the main component of microfilaments, and is commonly used to label F-actin rich structures such as pseudopod, and to observe the effects of treatments on actin cytoskeleton dynamics and cell motility [19–21]. FITC-labeled phalloidin staining and epifluorescence microscopy were therefore used to investigate the relationship between ARPC1A and the actin cytoskeleton. It demonstrated that suppression of ARPC1A expression inhibited the assembly of the filamentous actin cytoskeleton (Fig. 6K). In addition, we generated an ARPC1A-overexpressing PC-3 cell line for the in vivo experiments (Fig. 7A–C). We established a subcutaneous xenograft tumor model and a lung metastasis model in athymic nude mice to evaluate the effect of ARPC1A on tumor growth and metastasis. The results showed that there was no statistical difference on the size and the weight of xenograft tumors between the two groups (Fig. 7D–F). However, for the lung metastasis, nude mice in ARPC1A overexpression group had a higher degree than those in the control group (Fig. 7G–J).

### Glutamine metabolism promotes migration, invasion, and cytoskeleton formation of PCa cells through the up-regulation of ARPC1A

**Fig. 8:**
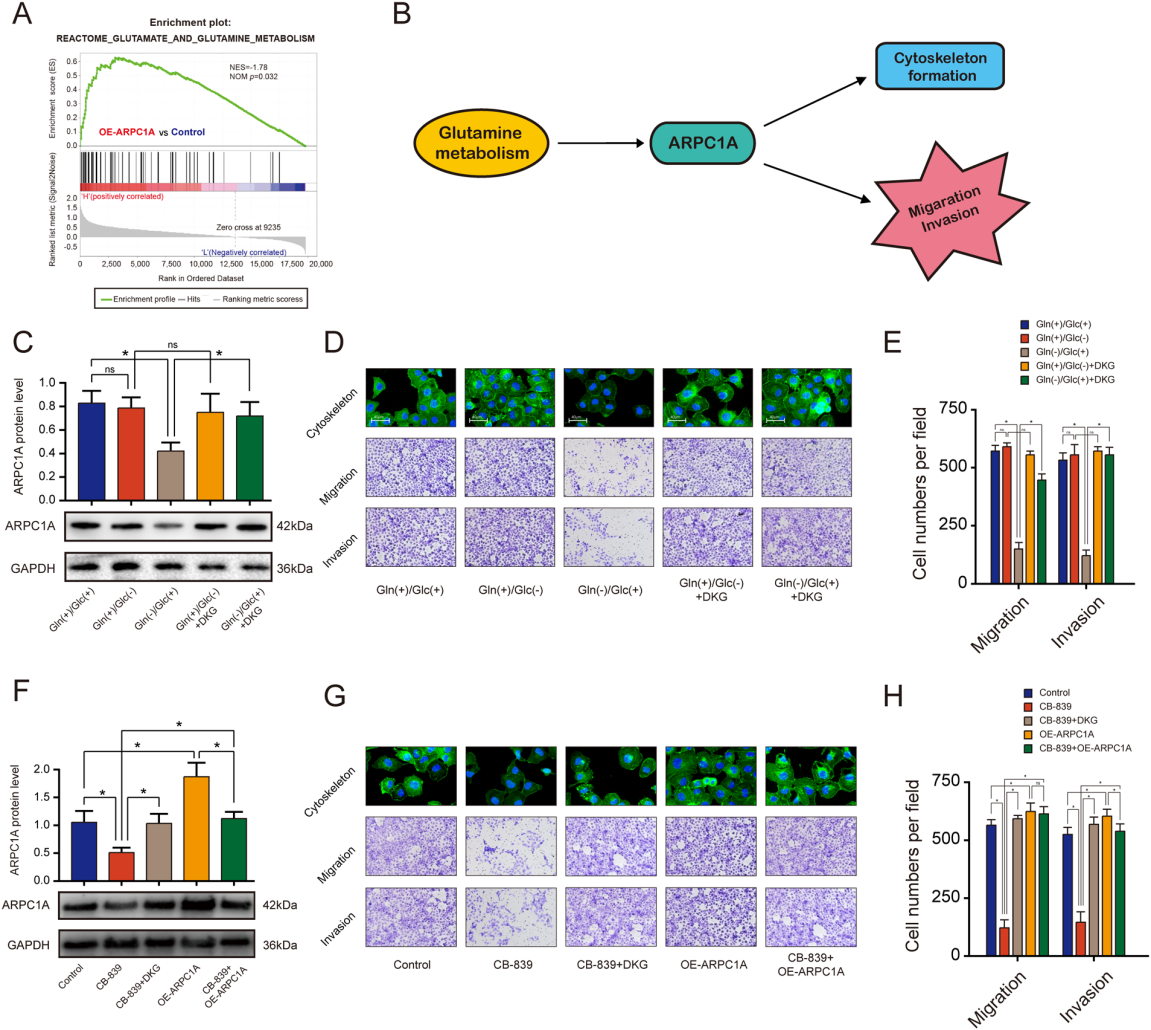
Glutamine metabolism promotes migration, invasion, and cytoskeleton morphology by up-regulating ARPC1A expression in PCa cells. **A** GSEA analysis revealed a significant positive association between high ARPC1A expression and glutamine metabolism. **B** Proposed model describing the roles of glutamine metabolism and ARPC1A in PCa. **C**–**E** ARPC1A expression, cytoskeletal morphology, and the migration and invasion potential of PC-3 cells cultured in different types of media. *: *P* < 0.05; *ns* no statistical significance, *Gln* glutamine, *Glc* Glucose, *DKG* dimethyl α-ketoglutarate. **F**–**H** ARPC1A expression, cytoskeletal morphology, and the migration and invasion potential of PC-3 cells under the indicated treatment conditions. *: *P* < 0.05; *ns* no statistical significance

Previous studies have shown that glutaminolysis regulates a variety of biological processes in a range of tumor types [22–24]. GSEA revealed a significant positive association between high ARPC1A expression and glutamine metabolism (Fig. 8A). We therefore hypothesized that glutamine metabolism affected the migration, invasion and cytoskeleton of PCa cells by regulating the expression of ARPC1A (Fig. 8B). To determine the effects of glutamine metabolism on PCa cells, we cultured the cells in glucose- or glutamine-deprived medium. Glutamine deprivation was found to completely inhibit the expression of ARPC1A, and to suppress migration, invasion and cytoskeleton formation in PCa cells. Glucose deprivation, however, had no effect (Fig. 8C–E). Since glutamate is converted into alpha-ketoglutarate (α-KG) to replenish the tricarboxylic acid cycle for energy production and anabolism, we tested whether the addition of cell-permeable dimethyl alpha-ketoglutarate (DKG) to the medium could rescue the phenotypes caused by glutamine deprivation. As predicted, DKG rescued these phenotypes, not only for cells cultured in glutamine-free culture medium (Fig. 8C–E), but also for those cultured in the presence of CB-839, a pharmacologic inhibitor of glutaminase (Fig. 8F–H). Additionally, it revealed that the inhibitory phenotypes caused by suppression of glutamine metabolism could be rescued upon elevated expression of ARPC1A, which indicated that glutamine metabolism was located in the upstream of ARPC1A and promoted migration, invasion and cytoskeleton formation of PCa cells via up-regulating the expression of ARPC1A (Fig. 8F–H).

## Discussion

To our knowledge, this study represents the first analysis of the relationship between ARPC1A and PCa. Our results demonstrated that ARPC1A is highly expressed in PCa tissues and is positively associated with malignant clinicopathologic characteristics, suggesting that this protein may be a potential prognostic factor for the progression of PCa. Further analysis using the Cox proportional hazard regression model demonstrated that patients with increased ARPC1A expression had a 1.581-fold greater risk of BCR after RP, when compared with those without elevated ARPC1A expression. Analysis of OS or survival using BCR as the endpoint, showed that ARPC1A was a good independent predictor of survival, demonstrating high sensitivity and specificity.

ARPC1A is a member of the ARP2/3 complex family [25]. The ARP2/3 complex was originally identified in Acanthamoeba and consists of seven proteins [26, 27]. It plays an essential role in lamellipodia-mediated cell migration, by virtue of its ability to generate branched actin filament networks, and this process is important for cancer invasion and spread [28, 29]. Kiuchi et al. demonstrated that ARP2/3 is involved in the formation of pseudopodia, as well as the movement of bladder cancer cells [30]. In glioma, cytoskeleton remodeling regulated by ARP2/3 has also been shown to be an important prerequisite for tumor metastasis and disease progression [31]. Additionally, it has been reported that components of the ARP2/3 complex are highly expressed in various tumor types, including gastric, breast, rectal, and lung cancers. And it is believed that the overexpression of these proteins promotes the migration and invasion of tumor cells and is a cause of poor patient prognosis in these diseases [32, 33]. However, the importance of ARPC1A in PCa cells and its role in the metastasis of PCa has not previously been established.

In this study, we performed additional functional assays to examine the role of ARPC1A in PCa cells. In vitro experiments demonstrated that ARPC1A knockdown inhibited the migration and invasion of PCa cells, but had no effect on cell proliferation and cell cycle progression. The results were consistent with those of the in vivo experiments. These findings suggest that elevated ARPC1A expression may increase the metastatic phenotype of PCa cells by increasing their motile behavior. The formation of invasive pseudopodia is dependent upon actin polymerization and dynamic cytoskeletal rearrangements [29]. We hypothesized that ARPC1A is involved in the formation of the cytoskeleton. Findings from our immunofluorescence assays suggested that actin filaments were less abundant in ARPC1A-knockdown PCa cells, when compared with control cells, an observation that supported our hypothesis. Liu et al. have also reported that glioma cells lose lamellipodia and cell polarity after treatment with CK666, a specific ARP2/3 inhibitor [31]. Our results indicate that ARPC1A may be a potential therapeutic target for PCa cell metastasis.

Glutamine is the most abundant amino acid in mammalian plasma. There is growing evidence to suggest that induction of glutaminolysis is a key step in the metabolic reprogramming that is necessary to fuel cancer cell growth [23, 34]. Bioinformatics analysis performed in our study revealed that high ARPC1A expression is associated with glutamine metabolism. We hypothesized that glutamine metabolism pomotes the expression of ARPC1A, and thereby migration, invasion and cytoskeleton formation in PCa cells. To determine the effects of glutaminolysis on PCa cells, glutamine-deprived culture medium and CB-839, a pharmacologic inhibitor of glutaminase, were used. These experiments revealed a suppression of migration, invasion and cytoskeleton formation, as well as a decreased cellular expression of ARPC1A, in PCa cells cultured in glutamine-deprived medium or medium supplemented with CB-839. However, the inhibitory phenotypes caused by glutamine deprivation could be rescued upon addition of DKG to the medium or following the forced upregulation of ARPC1A expression. These results demonstrate that ARPC1A works as a bridge between glutamine metabolism and downstream cellular phenotypes associated with tumor invasion and spread. Actually, we mainly used AR-negative PC-3 and DU-145 for in vitro experiments, so it is indeed unclear whether ARPC1A plays a similar role in AR-positive prostate cancer cells, which is one of the main limitations of this study.

## Conclusion

In summary, this study integrated data from PCa samples from our center with those from the TCGA database in an attempt to identify a potential biomarker of malignant PCa, using a bioinformatic-based approach. Further investigation for clinical significance and biological function revealed that ARPC1A was a good independent predictor for poor prognosis in PCa, and that this protein promoted cytoskeleton formation, and the migration and invasion of PCa cells. Glutamine metabolism was identified as an upstream regulator of this process. Therefore, inhibition of ARPC1A may provide a therapeutic strategy for the treatment of PCa.

## Supplementary Information

**Additional file 1: Figure S1. **WGCNA analysis based on the PCa samples from the TCGA database. **A** Analysis of the scale-free fit index for various soft-thresholding powers. **B** Analysis of the mean connectivity for various soft-thresholding powers. **C** Histogram of connectivity distribution when β = 16. **D** Evaluation of the scale-free topology when β = 16. **E** The sample dendrogram and corresponding clinical characteristics; red: high, blue: low. **F** Cluster dendrogram of the 434 PCa samples with eligible data. **G** Distribution of average gene significances and associated errors for the modules associated with T stage in PCa. **H** Relationship between module membership and gene significance for T stage in the turquoise module. **I** Distribution of average gene significances and associated errors in the modules associated with N stage in PCa. **J** Relationship between module membership and gene significance for N stage in the turquoise module. **K** Heatmap of the correlation between module eigengenes and different clinical characteristics of PCa.

## Data Availability

The datasets used and/or analysed during the current study are available from the corresponding author on reasonable request.

## Abbreviations

PCa: Prostate cancer
ARPC1A: Actin related protein 2/3 complex subunit 1A
TMA: The tissue microarray
RP: Radical prostatectomy
TCGA: The cancer genome atlas
WGCNA: Weighted gene co-expression network analysis
GS: Gene significance
MS: Module significance
ME: Module eigengene
GO: Gene ontology
GSEA: Gene set enrichment analysis
FDR: False discovery rate
STRING: Search Tool for the Retrieval of Interacting Genes
PPI: Protein–protein interaction
SiRNA: Short interfering RNA
qRT-PCR: Quantitative real-time polymerase chain reaction
WB: Western blotting
BCR: Biochemical recurrence (BCR)
ROC: Receiver operating characteristic
AUC: Area under the curve
OS: Overall survival
α-KG: Alpha-ketoglutarate
DKG: Dimethyl alpha-ketoglutarate
