# Supplementary figures and images for: ARPC1A correlates with poor prognosis in prostate cancer and is up-regulated by glutamine metabolism to promote tumor cell migration, invasion and cytoskeletal changes
Source: Cell Biosci. 2023 Feb 22;13:38. doi: 10.1186/s13578-023-00985-w (PMC9945620; doi:10.1186/s13578-023-00985-w)

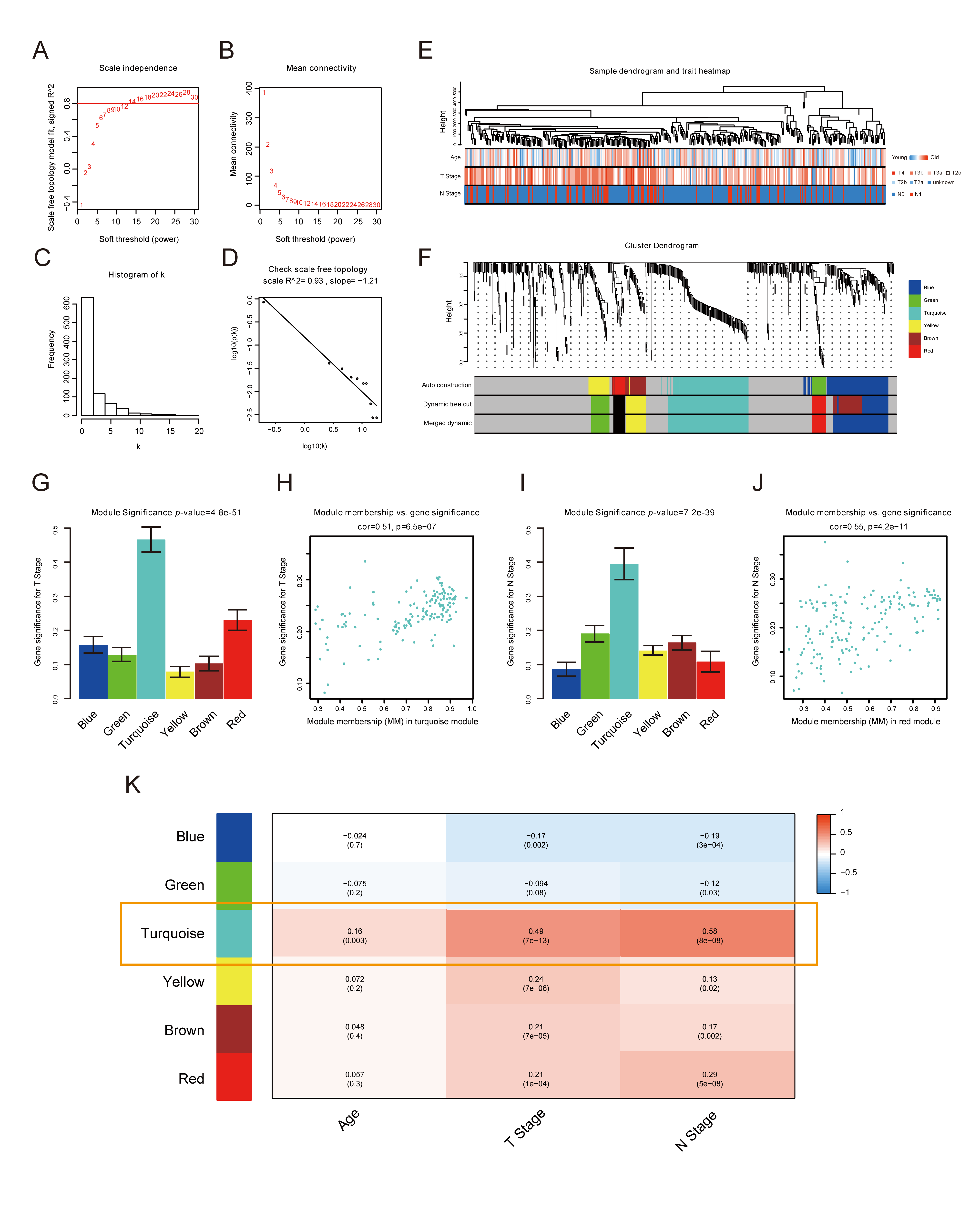

Supplement: Supplementary file 1 — Additional file 1: Figure S1. WGCNA analysis based on the PCa samples from the TCGA database. A Analysis of the scale-free fit index for various soft-thresholding powers. B Analysis of the mean connectivity for various soft-thresholding powers. C Histogram of connectivity distribution when β = 16. D Evaluation of the scale-free topology when β = 16. E The sample dendrogram and corresponding clinical characteristics; red: high, blue: low. F Cluster dendrogram of the 434 PCa samples with eligible data. G Distribution of average gene significances and associated errors for the modules associated with T stage in PCa. H Relationship between module membership and gene significance for T stage in the turquoise module. I Distribution of average gene significances and associated errors in the modules associated with N stage in PCa. J Relationship between module membership and gene significance for N stage in the turquoise module. K Heatmap of the correlation between module eigengenes and different clinical characteristics of PCa. [file 13578_2023_985_MOESM1_ESM.tif]
